# Supplementary material for: Splice-Junction-Based Mapping of Alternative Isoforms in the Human Proteome
Source: Cell Rep. Author manuscript; Available in PMC 2020 Jan 15. (PMC6961840; doi:10.1016/j.celrep.2019.11.026)
Supplement: 2 [file NIHMS1546469-supplement-2.zip › DF1/PXD006675/PulmonaryArtery/PulmonaryArtery_8_PALLD_LLGADSATVFNIQEPEEETANQIYWFK.pdf]

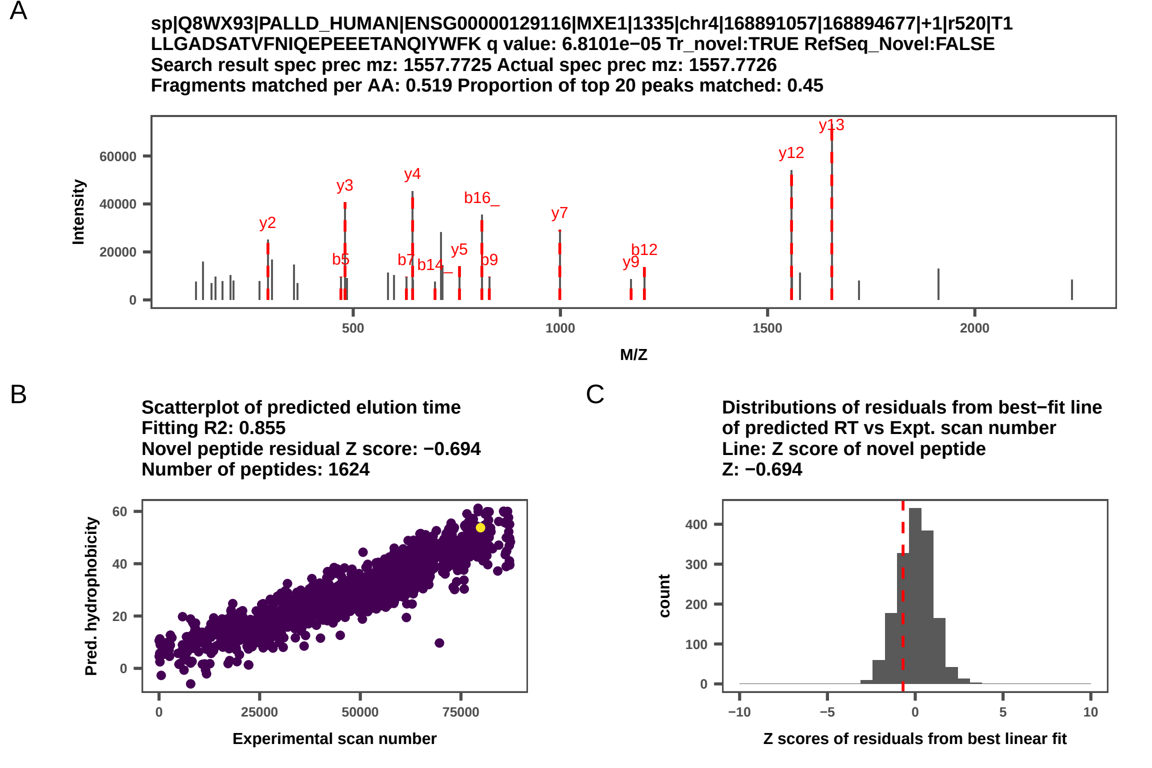
A
sp|Q8WX93|PALLD_HUMAN|ENSG00000129116|MXE1|1335|chr4|168891057|168894677|+1|r520|T1
LLGADSATVFNIQEPEEETANQIYWFK q value: 6.8101e−05 Tr_novel:TRUE RefSeq_Novel:FALSE
Search result spec prec mz: 1557.7725 Actual spec prec mz: 1557.7726
Fragments matched per AA: 0.519 Proportion of top 20 peaks matched: 0.45
Intensity
60000
40000
20000
0
y2
y3
b5
y4
b7
b14_
y5
b16_
b9
y7
y9
b12
y12
y13
500
1000
1500
2000
M/Z
B
Scatterplot of predicted elution time
Fitting R2: 0.855
Novel peptide residual Z score: −0.694
Number of peptides: 1624
Pred. hydrophobicity
60
40
20
0
0
25000
50000
75000
Experimental scan number
C
Distributions of residuals from best−fit line
of predicted RT vs Expt. scan number
Line: Z score of novel peptide
Z: −0.694
count
400
300
200
100
0
−10
−5
0
5
10
Z scores of residuals from best linear fit
